# Thematic Exordium for Special Issue “Density Functional Theory Application on Chemical Calculation”

**DOI:** 10.3390/ma16072904

**Published:** 2023-04-06

**Authors:** Denis V. Chachkov, Oleg V. Mikhailov

**Affiliations:** 1 Kazan Department of Joint Supercomputer Center of Russian Academy of Sciences—Branch of Federal Scientific Center “Scientific Research Institute for System Analysis of the RAS”, Lobachevskii Street 2/31, 420111 Kazan, Russia; 2 Department of Analytical Chemistry, Certification and Quality Management, Kazan National Research Technological University, K. Marx Street 68, 420015 Kazan, Russia

The history of quantum chemistry dates back to 1926, when the German physicist Erwin Schrödinger, in his classical works [1,2], formulated the so-called wave equation that describes the state of an electron in an atom and later became the basis for theoretical chemistry. This equation was based on the idea of the French physicist Louis de Broglie proposed exactly 100 years ago (in 1923) and published in early 1925 [3], according to which this elementary particle, along with its corpuscular properties, also has wave properties, i.e., corpuscular-wave dualism. In [1,2], the solution of this equation for the simplest of all atoms, the hydrogen atom, was also presented within the framework of which an interpretation of three quantum numbers characterizing the state of an electron in an atom was subsequently given, namely, the main (*n*), orbital (*l*) and magnetic (*m*) numbers. Although almost 100 years have passed since the publication of these works, the exact solution of this equation was only obtained for a given atom and so-called “hydrogen-like atoms” containing a nuclide with a charge Z and a single electron (it would be more correct to say ions since any of these particles has a positive charge (Z-1)^+^, Fe Li^2+^, O^7+^, P^14+^). However, it is precisely from these solutions that the traditional forms of atomic *s*-, *p*-, *d*- and *f*-orbitals follow, the images of which can now be found in any textbook of chemistry and sets out the theoretical foundations of this science. The reason why the exact solution of the above wave equation has not yet been obtained even for the next atom after hydrogen, namely He, is directly related to the problem of solving the so-called “many-body problem”, which consists of determining the relative motion of three or more bodies (material points) interacting according to Newton’s law of gravity. Unlike the two-body problem, in the general case, the problem does not have a solution in the form of finite analytical expressions even in the case of three bodies; only separate exact solutions for special initial velocities and coordinates of objects are known (at present, there are about 1000 such solutions) [4]. However, even if a general exact solution of this problem is found, it will not allow us to obtain an exact solution of the Schrödinger equation, since in the case of interaction between atoms, there are other factors that are absent in the many-body problem, namely, electrostatic and magnetic interactions. For molecules, the situation turns out to be immeasurably more complicated, and there is no possibility of an exact solution of the wave equation.

The simplest version of the chemical bond that occurs between the atoms of chemical elements is a two-center, two-electron bond. In this regard, it is quite natural that the first quantum mechanical description of the two-electron bond was the theoretical calculation of the simplest of all possible molecules, the hydrogen molecule H_2_, using the method of valence bonds, carried out by the German physicists Walter Heitler and Fritz London in 1927 [5]. This method was based on the hypothesis that when a molecule is formed from atoms, the latter largely retain their electronic configuration, and the binding of atoms is achieved as a result of the exchange of electrons between them and the pairing of the spins of two electrons located in the atomic orbitals of the initial atoms. The calculation carried out by these German physicists turned out to be very significant in terms of its results in the development of quantum chemistry; in particular, it was shown that the chemical bond in the hydrogen molecule is carried out precisely by a pair of electrons. This work served as a kind of prelude to the creation of a comprehensive theory of chemical bonding in the 1930s, called the “valence bond method”, which covered almost all molecules—from small to large, from the simplest hydrocarbons to metal complexes, as well as some solid bodies. The obvious (moreover, fundamental) drawback of this theory, however, was that, within its framework, the set of existing chemical bonds in each molecule was represented as a set of only two-center, two-electron bonds, which in the general case did not correspond to reality (because it was later experimentally proven that electrons are not localized between two atoms, but are delocalized within the framework of the nuclide skeleton of the molecule). In addition, other inconsistencies with experimental data were also found. The most significant of them (which was directly evident) was that, according to this method, the dioxygen O_2_ molecule should be diamagnetic, while the experimental data indicated that it is paramagnetic. The ability of a number of metal ions to form complexes with the same coordination number (C.N.) but with a different geometry (for example, Ni(II) with C.N. = 4 complexes, one part of which has a tetrahedral environment of ligands, e.g., [NiBr_4_]^2–^, while the other is planar rhombic or square, e.g., [Ni(CN)_4_]^2–^) was also incomprehensible. Using this method, it was not possible to explain the reason for the existence of those chemical compounds in which the number of chemical bonds, formed by an atom of a given chemical element with other atoms, which is greater than the number of its valence electrons allows. Therefore, when the ability of the so-called of inert gases [*p*-elements of group VIII (in the long-period version–XVIII)] to form fairly stable chemical compounds, a number of researchers were inclined to believe that in the process of formation of compounds of these elements, the valence electrons of their atoms are initially depaired to the next, higher energy level (namely, from the *ns*- and *np*-levels to the (*n*-1)*d*-level), and only then the unpaired electrons that appeared as a result of this process participated in the formation of two-center, two-electron bonds with other atoms according to the exchange mechanism of chemical bond formation. However, quantum chemical calculations within the framework of the theory of atomic structure have unequivocally shown that the energy required for such depairing is very large, and it could hardly be paid off by the energy that would be released as a result of the formation of the chemical bonds involving these electrons. For this reason alone, the possibility of realizing two-center, two-electron bonds in compounds formed by these chemical elements seems rather doubtful. However, an even more complicated situation is in the case of nitric acid HNO_3_, where nitrogen has an oxidation degree of +5, although the limiting number of chemical bonds formed by this element, within the framework of the valence bond method, cannot exceed the number of its valence orbitals (four—one 2*s* and three 2*p* atomic orbitals). The depairing of electrons from the 2*p*- (and even more so, 2*s*-) level of the nitrogen atom, even to the next energy level 3*s*, requires such high energy costs that there can be no talk of them, even in principle.

An alternative to the method of valence bonds was the Molecular Orbital–Linear Combination of Atomic Orbitals (MO-LCAO) method, which was introduced into scientific use in 1929 by Sir John Lennard-Jones in connection with the description of the bond in diatomic molecules, containing chemicals from the first row of the periodic system (i.e., 2*s*- and 2*p*-elements) and which is currently dominant among all other theoretical methods for describing the structure of molecules. Compared to the valence bond method, the MO-LCAO method has the following advantages:

- provides an interpretation of the chemical bond in hypervalent compounds (which are compounds formed by *p*-elements of group VIII (XVIII in the long-period version of the periodic table of D.I. Mendeleev, for example XeF_6_) within the framework of the concept of the formation of three-center, four-electron bonds;
- able to explain the formation of a chemical bond in electron-deficient molecules (e.g., diborane B_2_H_6_), molecular radicals (e.g., nitrogen monoxide NO), molecular ions (e.g., nitrosyl NO^+^, hydrazinium [N_2_H_5_]^+^, dioxygenyl O_2_^+^) based on the model of a three-center, two-electron bond;
- considers the hydrogen bond as a special case of the covalent bond, namely through the model of electron density delocalization and the formation of three-center, four-electron bonds (for example, –H•••[F–H•••F]– in the H_2_F_2_ molecule);
- allows us to quantitatively calculate a number of macroscopic parameters, the origin of which is associated with the specifics of molecules, primarily spectral and magnetic, due to the presence of an adequate mathematical apparatus.

The current methods of quantum chemistry can be divided into three groups: non-empirical, semi-empirical and methods based on the density functional theory. Non-empirical methods are often referred to as “ab initio”, which means “from first principles”. In such methods, any experimental data or parameters obtained from experimental data are not used at all. In contrast, semi-empirical calculation methods use parameters directly borrowed from experiments or selected in such a way that the calculation of a certain set of reference compounds best reproduces the physicochemical properties of these compounds. Density functional methods, the most widely used in recent decades for quantum chemical research, occupy an intermediate position, since they use a number of parameters that approximate the results of theoretical calculations of the properties of an electron gas.

The density functional theory (DFT) is based on the idea of the electron density in the ground state, the distribution of which is described by the one-particle Schrödinger equation. The predecessor of this theory was the model of the electronic structure of a many-body system using the semiclassical approximation authored by L. Thomas and E. Fermi [6,7]. Within the framework of this model, the energy of an atom in the ground state was represented as the sum of its kinetic energy in the form of an electron density functional, and the potential energy of interaction of electrons with the nucleus and with each other, which was also expressed in terms of electron density. In this case, however, the exchange interaction was not taken into account, and although in 1928 P. Dirac refined the energy functional in this model by adding a term in the form of an electron density functional describing the exchange interaction, in many cases this model could not give adequate descriptions of the electronic structure of the objects being described. The main source of error here was the expression for the kinetic energy, which leads to an error in the calculation of the exchange energy; moreover, the electron correlation energy was not taken into account in this model. A reliable theoretical justification for it was given only with the formulation of two Hohenberg–Kohn theorems [8]. According to the first of these theorems, the properties of the ground state of a many-electron system are determined only by the electron density, which depends on three coordinates; therefore, this theorem reduces the problem of describing a many-electron system of *N* electrons with 3*N* spatial coordinates to describing the electron density functional with three coordinates. According to the second theorem, the energy of the electron subsystem, written as the electron density functional, has a minimum value equal to the energy of the ground state (essentially, this is nothing but the well-known variational principle of quantum mechanics as applied to the density functional). As a rule, the DFT method is used in conjunction with the Kohn–Sham formalism, in which the problem of describing several interacting electrons in a static external field (atomic nuclei) is reduced to a simpler problem of independent electrons that move within some effective potential, including the static potential atomic nuclei, as well as taking into account the Coulomb interactions (in particular, the exchange interaction and electron correlation) [9]. It is the description of these two interactions that constitutes the main difficulty of the density functional theory method in the Kohn–Sham formulation. The simplest approximation here is the local density approximation, based on an exact calculation of the exchange energy for a spatially homogeneous electron gas, which can be performed within the framework of the Thomas–Fermi–Dirac model, and from which the correlation energy of the electron gas can also be obtained. In this regard, it should be noted that, initially, the Hohenberg–Kohn theorems were formulated only for the ground state of an electronic system in the absence of a magnetic field; however, they can be generalized by introducing a time dependence, and thus this formalism is also used to calculate the excited states of the system [10].

The DFT formalism, however, breaks down in the presence of a magnetic field. In this case, there is no one-to-one correspondence between the electron density and the external potential associated with the presence of nuclides. Attempts to generalize the formalism to take into account the effects associated with the magnetic field have led to two different versions of DFT, namely, the density functional theory with the current density vector taken into account, and the density functional theory with the magnetic field taken into account. In both cases, the functional of the exchange-correlation energy is generalized and becomes dependent not only on the electron density but also the magnetic field. In the first approach developed by Vignale and Rasolt [11], in addition to the electron density, the expression for the functional contains a parameter for the current density. In the second approach, described by Grayce and Harris [12], an additional parameter of the functional is the magnetic field strength, and the form of the functional depends on the form of the latter. For both methods, the calculation of the exchange-correlation energy beyond the local density approximation (or rather, its generalization to the case of a magnetic field) turned out to be extremely difficult.

DFT methods have been widely used for quantum-chemical calculations for more than 50 years. In a number of cases, the use of even a simple local density approximation gives quite satisfactory agreement with the experimental data, and the computational complexity of the method is low compared to other approaches to the many-particle problem in quantum mechanics. However, for a long time, the method was not accurate enough to carry out quantum-chemical calculations of various chemical compounds; the state of affairs changed significantly only in the last decade of the 20th century, when there was a noticeable shift in the description of exchange and correlation interactions. At present, the DFT is the dominant approach in both the above areas of physics and chemistry, and the number of works in which this method has been used in one way or another is already in the many thousands. A significant number of such articles have been published at various times in MDPI Journals.

It seems to us that at this point in time, at least the following three problems can be identified, which are one way or another related to the practical implementation of the DFT method:

- the problem of obtaining the most accurate analytical expressions for the functionals of exchange energy and correlation energy. The exact analytical expressions for the functionals of the exchange and correlation energies are known only for a particular case, namely for the so-called “electronic gas”, consisting of randomly moving electrons. Additionally, although the existing approximate expressions for these functionals make it possible to calculate a number of physical quantities with sufficient accuracy, the problem of their improvement remains relevant (and, perhaps, the most significant for this method);
- the problem of the multiplicity of DFT variants used for quantum chemical calculations. At present, there are at least several dozen different functionals (both “pure” and, most often, hybrid ones) widely used in the framework of DFT in the calculation of various chemical compounds. These, in particular, are the functionals B3LYP, B3PW91, PBE, M06 and their various modifications. This problem of multiplicity is a consequence of the fact that the functionals are not systematically improved, and therefore it is impossible to unambiguously predict which functional will lead to a smaller error in the calculation. In general, at this point in time, the situation with DFT methods is such that it is impossible to estimate in advance the error in calculating the molecular and electronic structures of chemical compounds using this theory without comparing its results with data obtained using higher levels of theory or with experimental results. This is why, the choice of a specific functional, as well as the basis set, is carried out either on the basis of comparison with data obtained using ab initio methods or, typically, on the basis of the researcher’s own experience and published results of DFT calculations by other scientists for similar objects and reactions;
- the problem of using DFT to describe certain types of interactions, the consideration of which, in some cases, is absolutely necessary. Therefore, despite the progress in the development of this theory, the problems associated with its application to the description of intermolecular forces, primarily van der Waals forces and dispersion interactions, still remain unresolved. The difficulties in calculating the dispersion interaction within the framework of this theory make it unsuitable for those systems in which the dispersion forces are predominant (in particular, when considering the interaction between atoms of noble gases) or those systems in which the dispersion forces are of the same order as other interactions between atoms and/or molecules (in the case of molecules of organic substances). The solution to this problem is also the subject of modern research. For example, a special DFT functional was developed, including the B97D dispersion interactions [13] and later, a number of modernized wB97D and wB97XD hybrid functionals based on it. Additionally, in recent years, it has become possible to add empirical accounting for dispersion interactions to the calculation of many other modern functionals, as was done in the work [14].

Be that as it may, at present, quantum chemical calculations using various methods, and primarily DFT, have become an integral part of most chemical research. These calculations make it possible to study chemical processes in detail at the molecular level, determine ways to improve the required properties and characteristics of chemicals and chemical processes, thereby increasing the efficiency of experimental studies and contributing to scientific and technological progress in general. Wherein, as already mentioned above, it is necessary to use various approximations that make it possible to achieve sufficiently reliable results. The works of Kendall Houk Laboratories [15], who have been simulating organic reactions using DFT methods for more than 20 years, should also be specially noted here. They showed that hybrid DFT methods lead to very small errors (on the order of 0.5 kJ/mol) in the relative energies of transition state conformers due to significant error compensation, which makes it possible to use DFT methods to quantitatively predict the enantioselectivities of organic reactions.

Among all modern methods of quantum chemistry, density functional theory methods have the important advantage that they provide the optimal ratio between the accuracy/reliability of calculations and the amount of computational resources expended. Additionally, this benefit contributed to their wide distribution as a tool for theoretical research of various chemical compounds.

Despite the fact that at present there are a number of works indicating very significant prospects for the use of this quantum chemical method of calculation, it would be clearly premature to assert that its potential possibilities have already been fully disclosed. This Special Issue of *Materials* is designed to contribute at least to some extent to the further development and improvement of this method.

## Data Availability

Not applicable.
